# Injectable cinnamaldehyde–loaded ZIF-8/Gallic Acid–Grafted gelatin hydrogel for enhanced angiogenesis and skin regeneration in diabetic wound healing

**DOI:** 10.3389/fbioe.2025.1660821

**Published:** 2025-09-11

**Authors:** Bo Zhou, Chen Zhang, Sheng Dai, Jin Zhao, Huaiyu Li, Yanbin Peng, Yunfeng Chu, Zhong Chen, Haotian Qin, Hui Zeng

**Affiliations:** ^1^ Department of Hand, Foot & Vascular Surgery, Peking University Shenzhen Hospital, Shenzhen, China; ^2^ School of Clinical Medicine, Hubei University of Medicine, Shiyan, China; ^3^ College of Medicine, Institute of Biomedical Engineering, Key Laboratory of Advanced Technologies of Materials, Ministry of Education, Southwest Jiaotong University, Chengdu, China; ^4^ Department of Bone & Joint Surgery, National & Local Joint Engineering Research Centre of Orthopaedic Biomaterials, Peking University Shenzhen Hospital, Shenzhen, China

**Keywords:** diabetic wound healing model, injectable hydrogel, cinnamaldehyde, ZIF-8 nanoparticles, angiogenesis, MOF-based delivery system

## Abstract

**Background:**

Chronic diabetic wounds remain a major clinical challenge due to persistent ischemia, oxidative stress, and impaired angiogenesis. Injectable hydrogels capable of adapting to irregular wound beds and delivering bioactive cues offer promising therapeutic potential for enhancing tissue regeneration.

**Methods:**

We developed a multifunctional injectable hydrogel by incorporating cinnamaldehyde-loaded ZIF-8 nanoparticles (CA@ZIF-8) into a gallic acid–grafted gelatin (GGA) matrix, followed by transglutaminase-mediated crosslinking. The physicochemical characteristics, drug release behavior, and mechanical performance of the CA@ZIF-8/GGA hydrogel were systematically evaluated. *In vitro* assays using human umbilical vein endothelial cells (HUVECs) were conducted to assess cytocompatibility and angiogenic activity. A full-thickness skin wound model in streptozotocin-induced diabetic rats was employed to evaluate *in vivo* wound healing efficacy and biocompatibility.

**Results:**

The CA@ZIF-8/GGA hydrogel exhibited favorable injectability, enhanced mechanical strength, and sustained release of both cinnamaldehyde and Zn^2+^. *In vitro*, the hydrogel significantly promoted HUVEC proliferation, migration, and tube formation, accompanied by upregulated expression of CD31 and VEGF. *In vivo*, CA(0.6)@ZIF-8/GGA-treated wounds demonstrated accelerated closure, enhanced granulation tissue formation, increased neovascularization, and re-epithelialization compared with control groups. No histological abnormalities were observed in major organs, indicating good systemic biocompatibility.

**Conclusion:**

This study presents an injectable CA@ZIF-8/GGA composite hydrogel that effectively promotes angiogenesis and diabetic wound regeneration. The synergistic integration of MOF-based controlled release and polyphenol-enhanced bioactivity highlights its potential as a clinically translatable platform for chronic wound management.

## 1 Introduction

Diabetic foot ulcer (DFU) is one of the most common chronic complications of diabetes, typically resulting from peripheral neuropathy, local ischemia, and infection. It is characterized by chronic non-healing wounds, recurrent ulceration, secondary infections, and tissue necrosis (Uccioli et al., 2015). With the global prevalence of diabetes steadily rising, the incidence of DFU is also increasing. According to the International Diabetes Federation, the global diabetic population is projected to reach 780 million by 2045, with 19%–34% of patients expected to develop DFU, making it the leading cause of non-traumatic lower-limb amputations among diabetic individuals (Shao et al., 2022). However, current clinical treatments for DFU face significant limitations. Conventional debridement heavily relies on surgical expertise and lacks objective biological indicators, while negative-pressure therapy is costly and poorly tolerated. Moreover, topical antibiotics often fail to address the chronic inflammation and impaired tissue regeneration associated with DFU (Zeng et al., 2019). Therefore, it is urgent to develop innovative materials with favorable biocompatibility, local responsiveness, and multifunctional therapeutic properties to improve DFU management (Mallawarachchi et al., 2019).

In recent years, hydrogel-based dressings have emerged as promising candidates for chronic wound healing due to their soft, moist nature, injectability, drug-loading capability, and responsiveness to local wound microenvironments (Liang et al., 2021). Injectable hydrogels can conform to irregular wound shapes, form a biomimetic barrier to maintain hydration, prevent infection, and extend drug residence time (Tang et al., 2023). Moreover, stimuli-responsive hydrogels can sense changes in pH, reactive oxygen species (ROS), glucose, or protease levels within the wound environment, triggering intelligent drug release and enhancing wound healing, especially in diabetic wounds (Pan et al., 2022). In our previous work, we developed a gelatin-gallic acid conjugate (GGA) hydrogel that forms a stable three-dimensional network via phenol-metal coordination or enzyme-mediated crosslinking (Yin et al., 2024). This hydrogel demonstrated excellent gelation ability, biocompatibility, antioxidant, and anti-inflammatory properties, and has been successfully applied in bone defect and osteoarthritis models (Zhao et al., 2024). Nevertheless, single-component hydrogels still face challenges in DFU treatment, such as limited therapeutic efficacy, insufficient responsiveness, and poor angiogenic performance.

To overcome these limitations, we proposed incorporating cinnamaldehyde-loaded ZIF-8 nanoparticles (CA@ZIF-8) into the GGA hydrogel system to construct a multifunctional composite hydrogel capable of simultaneously regulating inflammation and promoting angiogenesis. ZIF-8, a widely studied zinc-based metal-organic framework (MOF), possesses high porosity, good biocompatibility, and pH-responsive drug release characteristics (Ge et al., 2022). In acidic wound environments, ZIF-8 can release Zn^2+^ ions, which are known to activate angiogenic signaling pathways such as PI3K/Akt and HIF-1α, thereby promoting neovascularization (Li Y. et al., 2023). Moreover, its imidazole-rich structure also contributes to antibacterial and antioxidant functions (Zhang et al., 2025). Cinnamaldehyde (CA), a natural aromatic aldehyde derived from cinnamon, exhibits broad-spectrum antibacterial, anti-inflammatory, and pro-angiogenic properties and has shown promising potential in wound repair (Guan et al., 2025). Its unique aldehyde group can modulate ROS levels and suppress NF-κB-mediated inflammation, thereby improving the wound microenvironment and accelerating the transition from inflammation to tissue regeneration (Liu et al., 2025). Importantly, integrating CA into the ZIF-8 framework offers a wound-responsive co-delivery strategy, in which Zn^2+^ and CA are released in a coordinated manner. This design combines the vascular-promoting capacity of Zn^2+^ with the inflammation-resolving effects of CA, while the porous MOF structure stabilizes CA and enhances its local bioavailability. Such complementary actions are anticipated to address the dual challenges of vascular deficiency and chronic inflammation in diabetic wounds, thereby producing more effective and timely healing outcomes than either component alone. Thus, the integration of CA@ZIF-8 into GGA hydrogel is expected to create a synergistic platform with improved mechanical integrity, intelligent release behavior, and multifunctional therapeutic effects.

In this study, we developed a novel injectable composite hydrogel by dispersing CA@ZIF-8 nanoparticles into the GGA precursor solution, followed by transglutaminase (TGase)-mediated crosslinking and metal–phenol coordination. We systematically characterized the physicochemical properties of the resulting CA@ZIF-8/GGA hydrogel, including microstructure, rheology, degradation behavior, and drug release profile. The biological performance of the hydrogel was evaluated through *in vitro* assays on human umbilical vein endothelial cells (HUVECs) to assess its cytocompatibility and angiogenic potential. Furthermore, the therapeutic efficacy and mechanism of action were explored under conditions simulating the diabetic wound microenvironment. This study presents a promising strategy for the treatment of DFU, offering a responsive, multifunctional hydrogel platform with high clinical translation potential.

## 2 Materials and methods

### 2.1 Materials

Zinc acetate (Zn(CH_3_COO)_2_, ≥99%) was purchased from Aladdin Reagent Co., Ltd. (Shanghai, China). Cinnamaldehyde (CA, ≥98%) was obtained from Sigma-Aldrich (St. Louis, MO, United States). GA, 1-ethyl-3-(3-dimethylaminopropyl) carbodiimide hydrochloride (EDC), and N-hydroxysuccinimide (NHS) were also purchased from Sigma-Aldrich. N,N-Dimethylformamide (DMF) was supplied by Macklin Biochemical Co., Ltd. (Shanghai, China). Microbial transglutaminase (TG, 200 U/g), derived from Streptoverticillium mobaraense, was obtained from Shanghai Yuanye Biotechnology Co., Ltd. (Shanghai, China).

### 2.2 ZIF-8 and CA-loaded ZIF-8 synthesis and characterization

ZIF-8 nanoparticles were synthesized by a simple precipitation method (Ding et al., 2023). Briefly, 100 μL of zinc acetate dihydrate solution (90 mg/mL) was mixed with 900 μL of 2-methylimidazole solution (311 mg/mL), followed by the addition of 200 μL of reverse osmosis (RO) water. The mixture was stirred vigorously for 5 min at room temperature. The resulting suspension was centrifuged at 12,000 rpm for 10 min, and the precipitate was washed twice with RO water. The final product was dried at 37 °C to obtain ZIF-8 nanoparticles. To prepare CA-loaded ZIF-8 (CA@ZIF-8), CA was introduced at different concentrations (150, 300, 600, and 1,200 μg/mL) into the reaction mixture during ZIF-8 synthesis, following the same procedure as described above.

The morphology and size of ZIF-8 and CA@ZIF-8 nanoparticles were examined by transmission electron microscopy (TEM, JEOL JEM-2100, Japan) and scanning electron microscopy (SEM, JSM-7041F, JEOL, Tokyo, Japan). For TEM analysis, samples were dispersed in ethanol, dropped onto carbon-coated copper grids, and dried at room temperature. For SEM imaging, dried powder samples were mounted on metal stubs using conductive carbon tape and sputter-coated with a thin layer of gold prior to observation. Hydrogel pore size from SEM. Pore openings on the hydrogel surface were measured from top-view SEM micrographs using ImageJ (images calibrated to the embedded scale bar). Diameters were obtained by manual delineation of individual pores and reported as representative values. Fourier-transform infrared (FTIR) spectra were recorded using a Nicolet 5700 (Thermo Fisher Scientific, Waltham, United States) in the range of 4,000–500 cm^−1^. Dried powder samples were mixed with KBr and pressed into pellets for measurement. The crystalline structures of the nanoparticles were analyzed by X-ray diffraction (XRD, X’pert PRO, PANalytical, Almelo, Netherlands) using Cu Kα radiation with a scanning range of 5°–70° (2θ). X-ray photoelectron spectroscopy (XPS) was performed using an AXIS Ultra DLD spectrometer (Kratos Analytical, United Kingdom) equipped with a monochromatic Al Kα X-ray source to analyze the surface elemental composition and chemical states. High-resolution spectra were recorded for C 1*s*, N 1*s*, O 1*s*, and Zn 2*p*, and peak deconvolution was carried out using XPS peak 4.1 software.

### 2.3 Gallic-grafted injectable hydrogel synthesis

GGA was synthesized following a previously reported protocol with minor modifications (Li G. et al., 2023). Briefly, gelatin (5 g, from porcine skin, Type A, CAS No. 9000-70-8, Sigma-Aldrich VETEC) was dissolved in 150 mL of deionized water at 40 °C. Separately, GA (1.7 g, 10 mmol) was dissolved in a 3:2 (v/v) mixture of deionized water and DMF (125 mL total volume). EDC (1.91 g, 10 mmol) and NHS (1.6 g, 13.9 mmol) were added to activate the carboxyl groups of GA under stirring at 25 °C for 1 h, adjusting the pH to 4.5. The activated GA solution was then added to the gelatin solution and stirred overnight at 40 °C. The resulting mixture was dialyzed against deionized water for 7 days and subsequently lyophilized to obtain the GGA polymer.

The synthesized CA@ZIF-8 nanoparticles were incorporated into the GGA hydrogel matrix to fabricate nanocomposite hydrogels. Briefly, GGA (10 wt%) was dissolved in deionized water at 40 °C to form a uniform hydrogel precursor solution. CA@ZIF-8 nanoparticles were dispersed in RO water via mild sonication and added to the GGA solution under continuous stirring to achieve final nanoparticle concentrations of 2 mg/mL. TG (10 U/mL) was then introduced to initiate enzymatic crosslinking. The mixture was poured into molds and incubated at 37 °C for 5 min to form hydrogels.

### 2.4 Characteristics of hydrogels

The sol-gel transition behavior of hydrogels was evaluated using the vial inversion method. For the torsion test, hydrogel samples (10 × 100 × 1 mm^3^) were clamped at both ends with tweezers and twisted 360° for 5 min. Sample morphology and integrity were recorded photographically. Tensile testing was conducted on rectangular hydrogel strips (10 × 100 × 1 mm^3^) using a universal testing machine (AG-IS, Shimadzu, Japan) at a stretching rate of 5 mm/min. For compression tests, cylindrical samples (8 mm diameter, 10 mm height) were compressed at a rate of 5 mm/min using the same machine. The compressive modulus was determined from the slope of the linear portion of the stress-strain curve, and the compressive strength was defined as the maximum stress value. Each test was repeated on four samples, and representative curves were shown. The gelation kinetics and viscoelastic properties of the hydrogels were evaluated using a HAAKE rheometer (Malvern, United Kingdom) at 37 °C. GGA precursor solutions were mixed with 0.5 wt% TG and CA@ZIF-8 nanoparticles. The mixtures were gently stirred and immediately loaded onto a 20 mm quartz plate with a 1 mm gap. Time sweep tests were conducted at 1% strain to monitor the sol–gel transition. Frequency sweeps were subsequently performed on fully crosslinked hydrogels over the range of 0.1–10 Hz to characterize their viscoelastic behavior.

The internal morphology of freeze-dried hydrogels was examined by SEM using the same instrument and sample preparation protocol as described for nanoparticle analysis. FTIR spectra were recorded using the same Nicolet 5700 spectrometer to analyze the functional groups in different hydrogel formulations. XRD patterns were collected on the same X’pert PRO diffractometer to evaluate the crystalline structure of the composites. XPS analysis was performed using the same AXIS Ultra DLD spectrometer, including high-resolution scans for C 1*s*, N 1*s*, O 1*s*, and Zn 2*p* to investigate surface elemental composition and chemical bonding states.

Cylindrical hydrogels (8 mm diameter, 10 mm height) were immersed in PBS (pH 7.4) at 37 °C for 21 days to assess degradation behavior. At days 1, 4, 7, and 14, samples were lyophilized and weighed. Mass retention was calculated as (W_1_/W_0_) × 100%, where W_0_ and W_1_ represent the dry weights before and after immersion, respectively.

For ion and drug release analysis, four hydrogels were incubated in 3 mL PBS, with the medium refreshed at each time point. Zn^2+^ concentrations were measured by ICP-OES (Agilent 730, United States), and CA levels were determined using UV–vis spectrophotometry (UV-2600, Shimadzu, Japan) at 290 nm, based on a PBS-derived standard curve. All experiments were conducted in triplicate.

### 2.5 Cell culture

Human umbilical vein endothelial cells (HUVECs) were obtained from Procell Life Science & Technology Co., Ltd. (Wuhan, China; catalog no. CP-H082) and cultured in endothelial cell medium (ECM; Procell) supplemented with 5% fetal bovine serum (FBS), 1% endothelial cell growth supplement (ECGS), and 1% penicillin-streptomycin. Cells were maintained in a humidified incubator at 37 °C with 5% CO_2_. HUVECs at passages 3–6 were used for all subsequent experiments. Mouse fibroblast L929 cells were purchased from Procell Life Science & Technology Co., Ltd. (Wuhan, China; catalog no. CL‐0137) and cultured in Dulbecco’s Modified Eagle Medium (DMEM; Gibco, USA) supplemented with 10% FBS and 1% penicillin‐streptomycin. Cells were maintained at 37 °C in a humidified incubator with 5% CO_2_, and passages 3‐8 were used for the experiments.

### 2.6 Live/dead staining

HUVECs were seeded onto hydrogel-coated 24-well plates at a density of 2 × 10^4^ cells/well and cultured in endothelial cell medium (ECM) supplemented with 10% FBS for 24 h. Live and dead cells were stained using Calcein-AM (2 μM) and propidium iodide (PI, 4 μM) (Beyotime, China) according to the manufacturer’s instructions. Fluorescence images were captured with an inverted fluorescence microscope (Nikon Ti2, Japan).

### 2.7 F-actin and DAPI staining

For cytoskeletal staining, HUVECs cultured on different hydrogels for 24 h were fixed with 4% paraformaldehyde for 15 min, permeabilized with 0.1% Triton X-100 for 10 min, and stained with rhodamine–phalloidin (50 μg/mL, Sigma) for 30 min at room temperature. Nuclei were counterstained with DAPI (5 μg/mL, Sigma) for 5 min. Fluorescence images were acquired using a confocal laser scanning microscope (Zeiss LSM 880, Germany) under identical exposure settings across all groups.

### 2.8 CD31 and VEGF immunofluorescence

After 24 h incubation with hydrogel extracts, HUVECs were fixed with 4% paraformaldehyde and blocked with 1% BSA for 1 h. Cells were incubated overnight at 4 °C with primary antibodies against CD31 (1:200, Abcam, ab28364) or VEGF (1:200, Abcam, ab46154), followed by Alexa Fluor–conjugated secondary antibodies (1:500, Invitrogen, A-11001/A-11008) for 1 h at room temperature. Nuclei were counterstained with DAPI (5 μg/mL, Sigma, D9542). Images were captured using a confocal microscope (Zeiss LSM 880, Germany) with identical exposure times for all groups. Fluorescence intensity was quantified using ImageJ software, and values were normalized to the control group (set as 1.0).

### 2.9 Migration assay

A Transwell assay was conducted using 8 μm pore-size inserts (Corning, United States). HUVECs (1 × 10^5^) suspended in serum-free medium were seeded in the upper chamber, and 600 μL of hydrogel extract medium was added to the lower chamber. After 12 h, migrated cells on the lower surface were fixed, stained with 0.1% crystal violet, and counted under a microscope in five random fields.

### 2.10 Tube formation assay

Matrigel (Corning, United States) was added to 96-well plates (50 μL/well) and incubated at 37 °C for 30 min. HUVECs (1 × 10^4^/well) were seeded in hydrogel extract–containing medium and incubated for 6 h. Tubular structures were imaged and quantified using ImageJ (Angiogenesis Analyzer plugin) for branch number and total tube length.

### 2.11 Animal model

All animal experiments were approved by the Institutional Animal Care and Use Committee of Shenzhen Peking University–The Hong Kong University of Science and Technology Medical Center (Approval No. 2025-525). Male Sprague–Dawley (SD) rats (8 weeks old, 220–250 g) were rendered diabetic by intraperitoneal injection of streptozotocin (STZ, 50 mg/kg) for five consecutive days. Rats with fasting blood glucose levels exceeding 16.7 mmol/L for three consecutive days were considered diabetic and included in subsequent experiments. Full-thickness wounds were created 4 weeks after STZ injection, by which time the diabetic state was stable. Blood glucose levels were monitored weekly throughout the wound healing observation period, and remained consistently above 16.7 mmol/L without significant fluctuations among groups.

### 2.12 Wound creation and treatment

Under anesthesia, a full-thickness circular skin wound (2 cm in diameter) was created on the dorsal area of each rat using a sterile biopsy punch. The animals were randomly assigned to four groups (n = 5): Control (PBS), GGA, ZIF-8/GGA, and CA(0.6)@ZIF-8/GGA. Each wound was topically treated with 200 μL of the respective hydrogel and covered with a sterile occlusive dressing (Tegaderm™).

### 2.13 Wound closure analysis

Wound healing was documented photographically on days 0, 4, 8, and 12 under standardized bright-field conditions (fixed camera–wound distance, identical illumination intensity, and locked exposure/white balance; a 1-cm scale bar was included in every image). Wound area was measured in ImageJ by manual tracing of the wound boundary and expressed as A_t_/A_0_ ×100%; data are reported as mean ± SD (n = 6 per group). A standardized healing score (0–4) was assigned based on macroscopic appearance, re-epithelialization, and hair regrowth, using the following rubric: 0 = no closure, moist surface/exudate; 1 = ≤25% closure with persistent exudate/necrosis; 2 = 26–50% closure with partial re-epithelialization; 3 = 51–75% closure with obvious re-epithelialization/granulation and minimal exudate; 4 = ≥90% closure with continuous epithelium, dry surface, minimal/no exudate, and initial hair regrowth. Full criteria are provided in Supplementary Table S1.

### 2.14 Biosafety evaluation

To evaluate systemic biocompatibility, major organs including the lung, liver, heart, brain, spleen, and kidney were harvested from each group on day 12 post-treatment. Tissues were fixed in 4% paraformaldehyde, embedded in paraffin, sectioned at 5 µm thickness, and stained with hematoxylin and eosin (H&E) using standard protocols. Histopathological examination was performed under a light microscope (20× and 40× magnification) in a blinded manner by a trained pathologist. The assessment focused on structural integrity, inflammatory infiltration, and potential tissue damage. No additional blood biochemical tests were conducted in this study.

## 3 Results

### 3.1 Characterization of synthesized ZIF-8 and CA-loaded ZIF-8 nanoparticles

**FIGURE 1:**
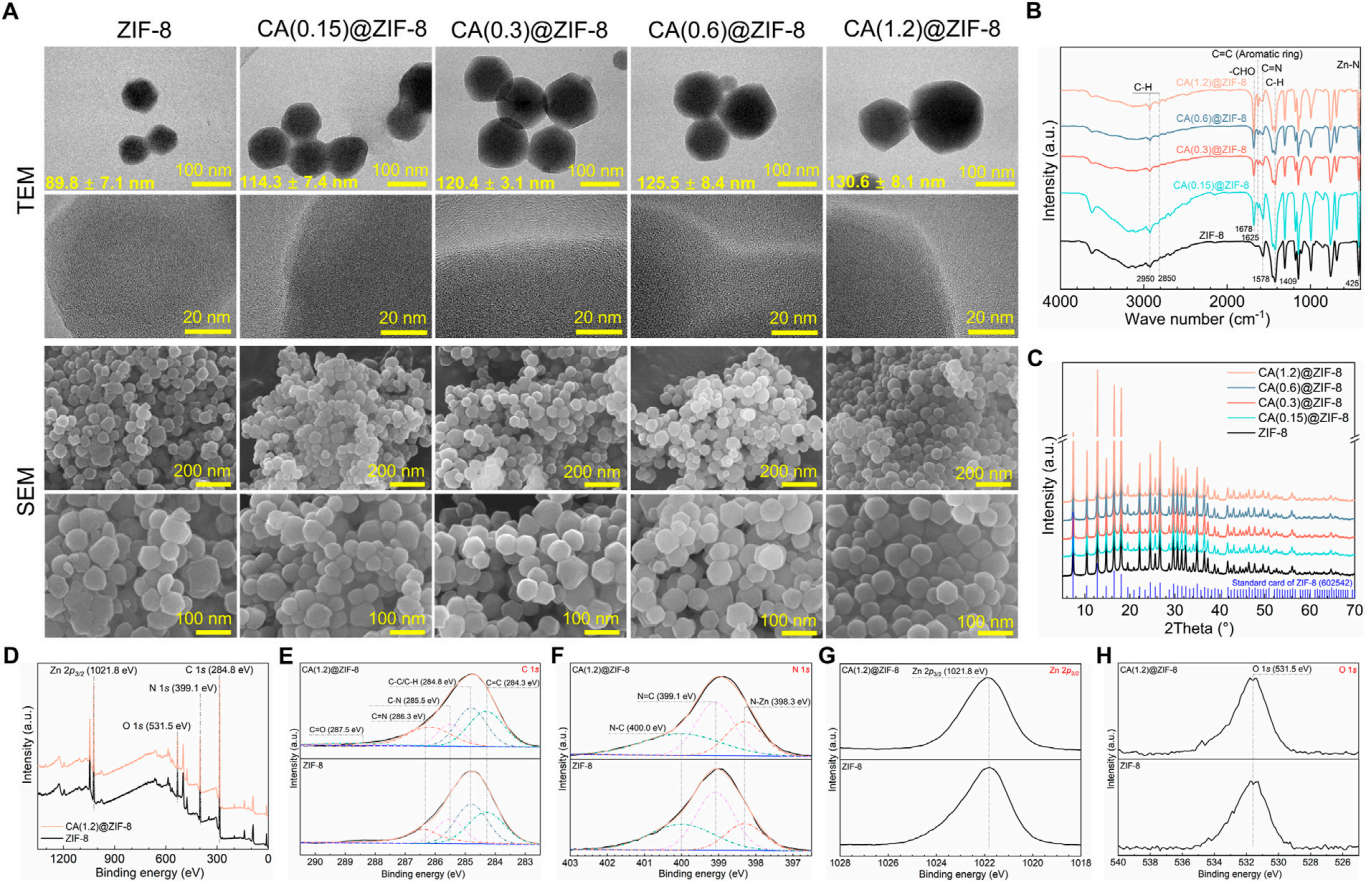
Morphology and physicochemical characterization of ZIF-8 and CA-loaded ZIF-8 nanoparticles. **(A)** TEM and SEM images of ZIF-8 and CA(x)@ZIF-8 with x = 0.15, 0.3, 0.6, and 1.2. Mean particle diameters (mean ± SD) are shown on the TEM images. Scale bars: top-row TEM = 100 nm; second-row TEM = 20 nm; third-row SEM = 200 nm; fourth-row SEM = 100 nm. CA(x) denotes the CA feed concentration during loading (g/mL). **(B)** FTIR spectra highlighting characteristic bands from the imidazolate linker and CA. **(C)** XRD patterns showing preserved ZIF-8 crystallinity after CA loading (reference pattern shown for comparison). **(D)** XPS survey spectra with major peak assignments: Zn 2*p*, N 1*s*, O 1*s*, and C 1*s* (charge correction to C 1*s* = 284.8 eV). **(E)** High-resolution C 1*s* spectra deconvoluted into C=C (≈284.3 eV), C–C/C–H (284.8 eV), C–N (≈285.5eV), C=N (≈286.3 eV), and C=O (≈287.5 eV). **(F)** High-resolution N 1*s* spectra deconvoluted into N–Zn (≈398.3 eV), N=C (≈399.1 eV), and N–C (≈400.0 eV). **(G)** High-resolution Zn 2*p*_3/2_ centered at 1,021.8 eV. **(H)** High-resolution O 1*s* spectra with maxima around 531.5 eV.

Figure 1 displays the materials characterization of ZIF-8 and CA-loaded ZIF-8 nanoparticles. As illustrated in Figure 1A, the TEM and SEM images revealed that pristine ZIF-8 nanoparticles exhibited a uniform polyhedral morphology with an average size of approximately 89.8 ± 7.1 nm. After loading CA, the particle size gradually increased with CA content, reaching up to 130.6 ± 8.1 nm for CA(1.2)@ZIF-8, while maintaining structural integrity. FTIR spectra (Figure 1B) confirmed the presence of characteristic peaks from both ZIF-8 and CA. Peaks at ∼1,578 cm^−1^ (C=N) and 425 cm^−1^ (Zn–N) are attributed to the imidazole ring and the coordination between Zn^2+^ ion and imidazole groups, respectively (Qian et al., 2022; Qian et al., 2023), indicating the successful formation of the ZIF-8 framework. After CA loading, new bands appeared at ∼1,678 cm^−1^ (–CHO) and ∼1,625 cm^−1^ (C=C, aromatic ring), confirming the incorporation of CA without disrupting the ZIF-8 structure. XRD patterns (Figure 1C) showed that all CA@ZIF-8 samples retained the crystalline structure of ZIF-8. XPS confirmed the elemental composition and surface states of ZIF-8 before and after CA loading. In the survey spectra (Figure 1D), both samples display C 1*s* (284.8 eV), N 1*s* (399.1 eV), Zn 2*p* (1,021.8 eV), and O 1*s* (531.5 eV) signals. The O 1*s* intensity is slightly higher for CA(1.2)@ZIF-8, consistent with the presence of oxygen-containing species introduced during loading and/or ubiquitous surface adsorbates. High-resolution C 1*s* spectra (Figure 1E) are well deconvoluted into four components located at 284.8 eV (C–C/C–H), ≈284.3 eV (C=C, aromatic/conjugated), ≈285.5 eV (C–N), and ≈286.3 eV (C=N), in agreement with the imidazolate linker and the aromatic framework. The N 1*s* spectra (Figure 1F) comprise N–Zn (≈398.3 eV), N=C (≈399.1 eV) and N–C (≈400.0 eV) components; the peak positions remain essentially unchanged after CA loading, indicating that the Zn–N coordination environment of ZIF-8 is preserved. The Zn 2p spectrum (Figure 1G) shows a Zn 2*p*_3/2_ peak centered at 1,021.8 eV, characteristic of Zn^2+^. Overall, the XPS results verify successful CA loading without disrupting the ZIF-8 coordination framework.

### 3.2 Mechanical performance, rheological properties, and degradation behavior

**FIGURE 2:**
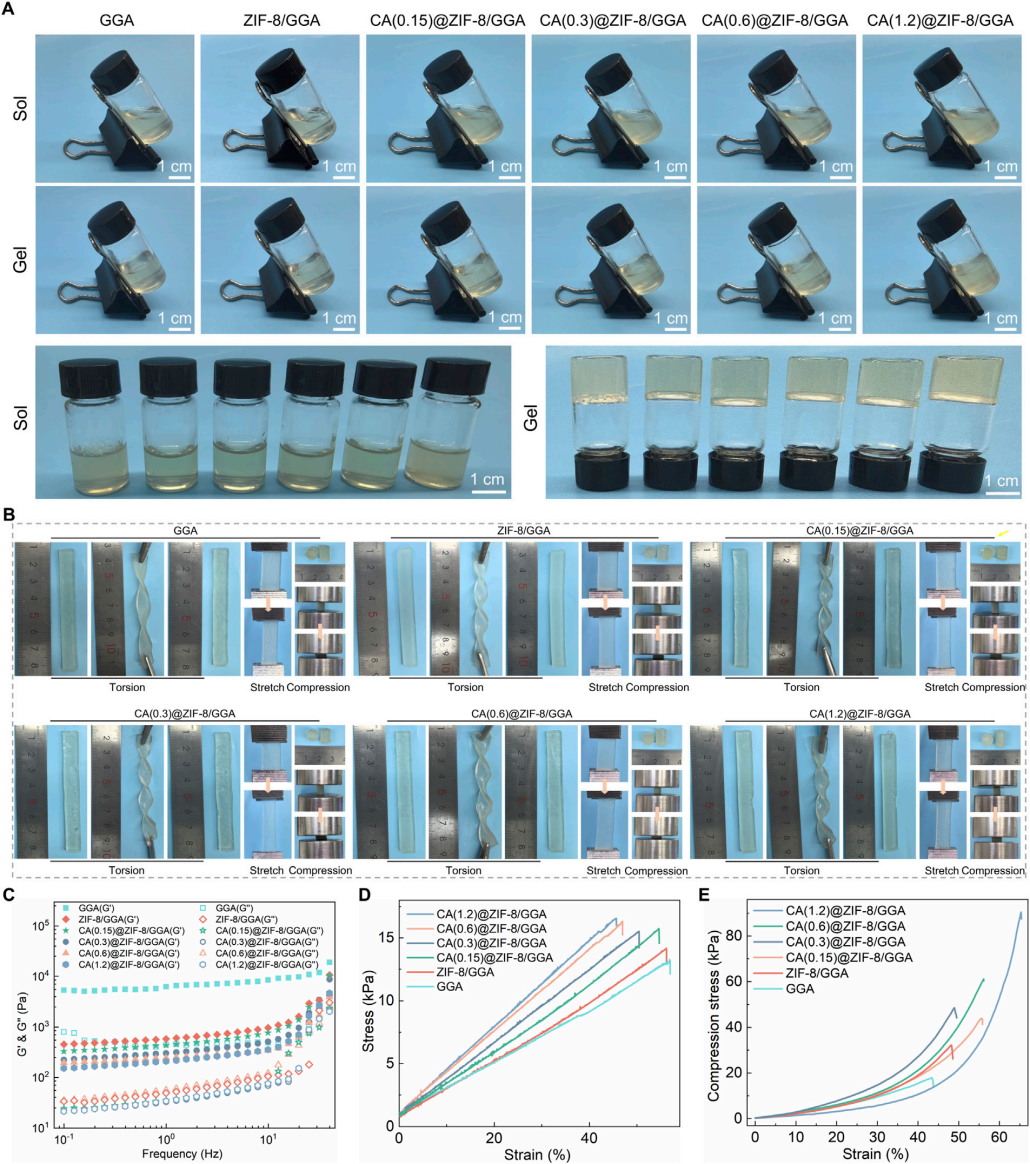
Visual gelation, macroscopic deformation, post-gel rheology and mechanics of CA(x)@ZIF-8/GGA hydrogels (x = 0.15, 0.3, 0.6, 1.2). **(A)** Inverted-vial photographs before (Sol) and after gelation (Gel) for GGA, ZIF-8/GGA and CA(x)@ZIF-8/GGA. Scale bars = 1 cm. **(B)** Macroscopic torsion, stretch, and compression tests with 1 cm rulers. Within the tested deformation/strain window, no visible fracture was observed for any formulation (representative images shown). **(C)** Frequency-sweep rheology (0.1–20 Hz, within the linear viscoelastic region) acquired after gelation, showing storage (G′) and loss (G″) moduli; G′ > G″ across the entire frequency range with no crossover, indicating elastic-dominant networks. The gelation time for the representative formulation is reported in the text (≈3 min after TGase addition, inverted-vial criterion). **(D)** Tensile stress–strain curves of hydrogels. **(E)** Compressive stress–strain curves of hydrogels. Colors/symbols are harmonized across panels as shown in the legends.

**FIGURE 3:**
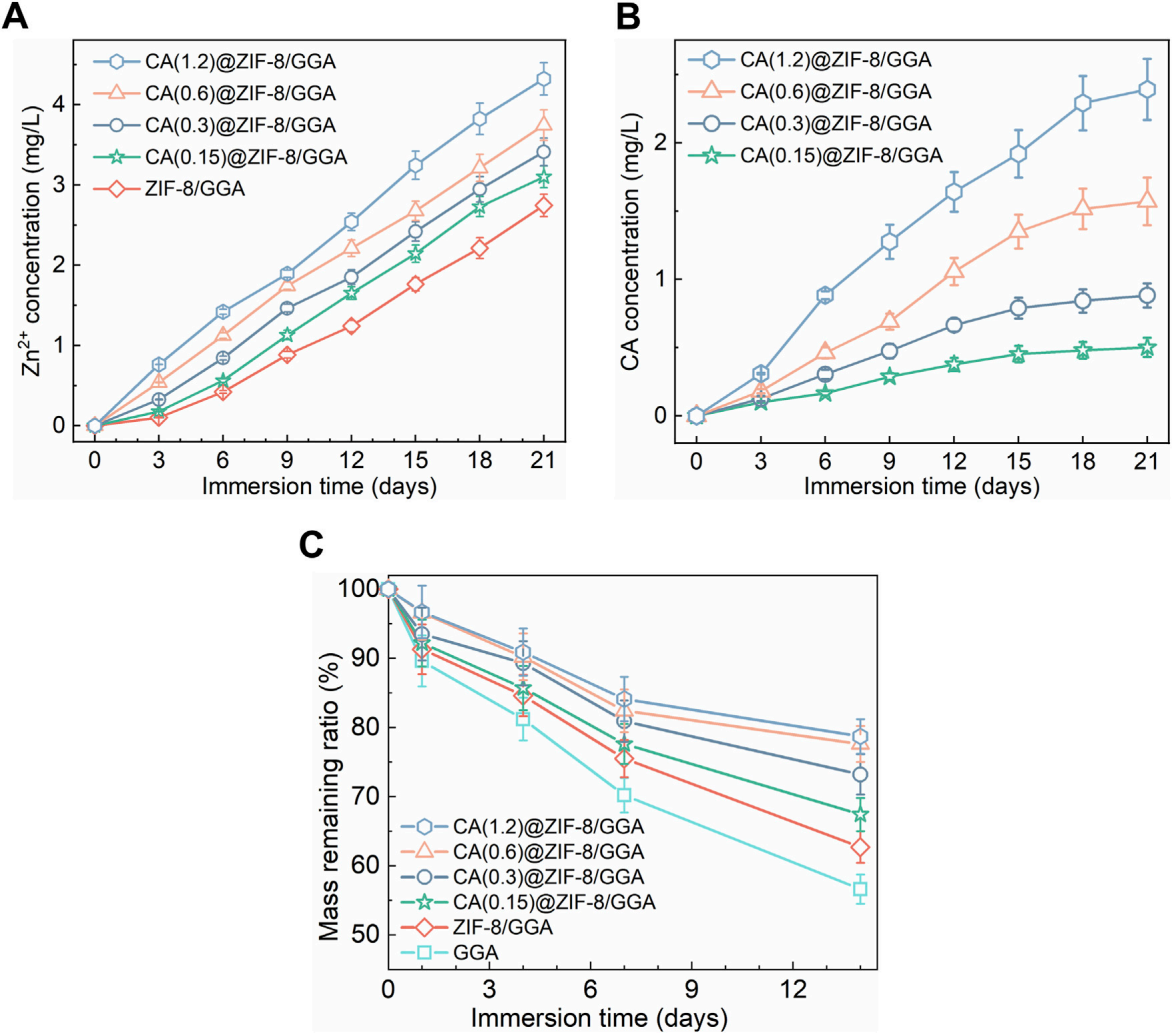
Ion/drug release and mass loss of CA(x)@ZIF-8/GGA hydrogels under sink conditions at 37 °C, (x = 0.15, 0.3, 0.6, 1.2). **(A)** Zn^2+^ release from ZIF-8/GGA and CA(x)@ZIF-8/GGA, presented as cumulative concentration in the release medium (mg/L). Quantified by ICP-OES. **(B)** CA release from CA(x)@ZIF-8/GGA, shown as cumulative concentration (mg/L). Quantified by UV-Vis spectroscopy at λ = 290 nm using calibration curves (R^2^ ≥ 0.99). **(C)** Mass remaining ratio (%) of hydrogels during incubation in buffer (pH 7.4) at 37 °C. Data are mean ± SD (n = 3). “Cumulative” indicates that concentrations at each time point include contributions from all prior intervals.

The sol–gel transition of GGA, ZIF-8/GGA, and CA(0.15, 0.3, 0.6, 1.2)@ZIF-8/GGA hydrogels was assessed by vial inversion, indicating successful gel formation across all formulations (Figure 2A). The resulting hydrogels exhibited good shape integrity after molding. Macroscopic deformation tests demonstrated the hydrogels’ ability to withstand torsion, stretching, and compression without fragmentation (Figure 2B). Rheological analysis (Figure 2C) showed that all hydrogel samples exhibited typical viscoelastic solid behavior, with storage modulus (G′) consistently higher than loss modulus (G″) across the tested frequency range. Notably, both G′ and G″ values increased with higher CA@ZIF-8 content, indicating enhanced gel stiffness and elasticity. In tensile tests (Figure 2D), the stress-strain curves demonstrated improved tensile strength and extensibility as CA@ZIF-8 concentration increased. Similarly, the compressive stress-strain curves (Figure 2E) revealed a marked increase in compressive resistance with higher filler content, suggesting that CA@ZIF-8 incorporation effectively reinforced the hydrogel network. Ion release studies showed a time-dependent and concentration-dependent release of both Zn^2+^ and CA (Figures 3A,B). Higher CA@ZIF-8 loading led to faster and more sustained release of Zn^2+^, while CA release exhibited an initial burst phase followed by a slower, sustained profile, with release rates positively correlated with CA content. Degradation analysis (Figure 3C) demonstrated a gradual decrease in hydrogel mass over 12 days, with faster degradation observed in samples containing higher CA@ZIF-8 content, suggesting that filler concentration influences hydrogel stability in PBS.

### 3.3 Characterization of hydrogel

**FIGURE 4:**
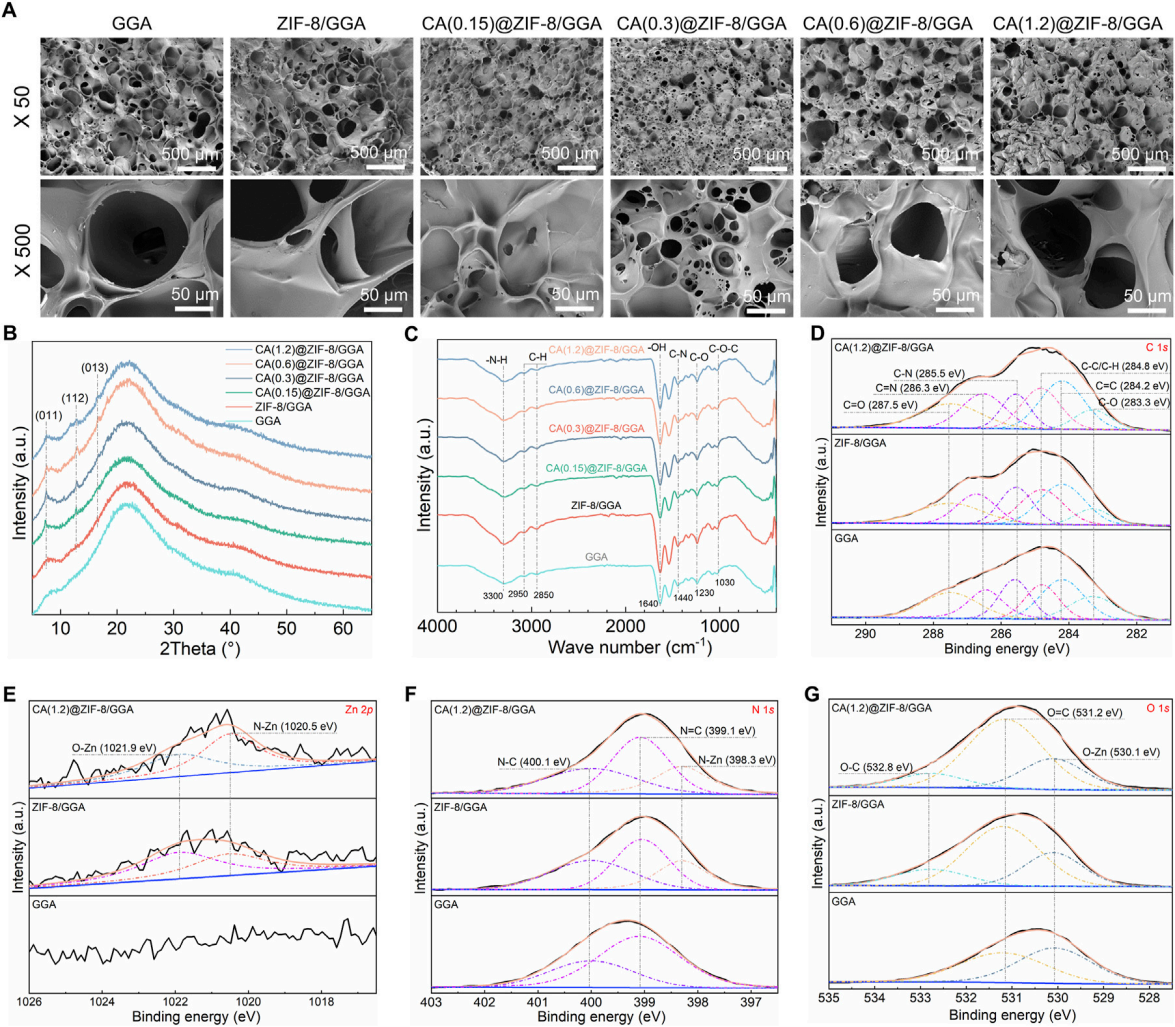
Morphology and physicochemical characterization of GGA-based hydrogels incorporating CA@ZIF-8. **(A)** Top-view SEM images of GGA, ZIF-8/GGA, and CA(x)@ZIF-8/GGA (x = 0.15, 0.3, 0.6, 1.2) at ×50 and ×500. Scale bars: 500 μm (top row) and 50 μm (bottom row). Pore openings were read from top-view SEM micrographs using ImageJ on scale-calibrated images (manual measurements). **(B)** XRD patterns showing characteristic reflections of ZIF-8 (e.g., (011), (112), (013)) retained after incorporation into the hydrogels. **(C)** FTIR spectra highlighting bands assigned to–NH/–OH, C–N/C=N of the imidazolate linker, aromatic C=C, and CA-related carbonyl vibrations. **(D)** High-resolution C 1*s* spectra deconvoluted into C–C/C–H, C=C, C–N, C=N, and carbonyl components (binding-energy values indicated on the plots). **(E)** Zn 2*p* region showing Zn 2*p*_3/2_ centered near ∼1,021.8 eV (spin-orbit partner 2*p*_1/2_ expected at ∼1,044.8 eV). **(F)** N 1*s* spectra resolved into N–Zn, N=C, and N–C components. **(G)** O 1*s* spectra with contributions assigned to Zn–O/–OH and O–C species, as labeled. All XPS spectra were charge-corrected to C 1*s* = 284.8 eV and fitted using a Shirley background with Gaussian–Lorentzian peak shapes. Colors/symbols are harmonized across panels; CA(x) denotes the CA feed concentration used during loading.

The internal microstructure of the freeze-dried hydrogels was observed by SEM (Figure 4A), showing that pore size decreased and the network became denser with increasing CA@ZIF-8 content, indicating the influence of nanoparticle incorporation on hydrogel architecture. XRD patterns (Figure 4B) revealed that all composite hydrogels maintained the characteristic peaks of ZIF-8, with intensities gradually increasing as the CA@ZIF-8 content increased, suggesting enhanced crystalline phase retention. FTIR spectra (Figure 4C) showed the characteristic absorption bands of GGA, ZIF-8, and CA, confirming the coexistence of the three components in the composite hydrogels. XPS analysis further confirmed compositional changes: the C 1*s* spectra (Figure 4D) showed increased intensity of C=O and C-N components; Zn 2*p* peaks (Figure 4E) were present in ZIF-8-containing samples but absent in GGA; the N 1*s* spectrum (Figure 4F) showed the emergence of N-Zn peak; and the O 1*s* spectrum (Figure 4G) exhibited enhanced O=C and O-Zn components with increasing CA@ZIF-8 incorporation, confirming the successful integration of the nano-fillers and their interaction with the hydrogel matrix.

### 3.4 Cytocompatibility assessment with L929 fibroblasts

The cytocompatibility of the hydrogels was further verified using L929 fibroblasts (Supplementary Figure S2). Live/dead staining (Supplementary Figure S2A) showed that most cells remained viable in the GGA and ZIF-8/GGA groups, with a progressive increase in live cell density upon CA incorporation. In particular, CA(0.6)@ZIF-8/GGA exhibited the highest proportion of live cells and minimal dead cells, whereas CA(1.2)@ZIF-8/GGA induced marked cytotoxicity with a large number of dead cells. Consistently, quantitative analysis by CCK-8 assay (Supplementary Figure S2B) confirmed that CA(0.6)@ZIF-8/GGA significantly promoted fibroblast viability, while excessive CA loading led to a sharp decline. These results demonstrate that CA(0.6)@ZIF-8/GGA provides an optimal balance of cell compatibility and bioactivity in fibroblasts, supporting its potential application in skin tissue repair.

### 3.5 *In vitro* angiogenesis activity of hydrogels

**FIGURE 5:**
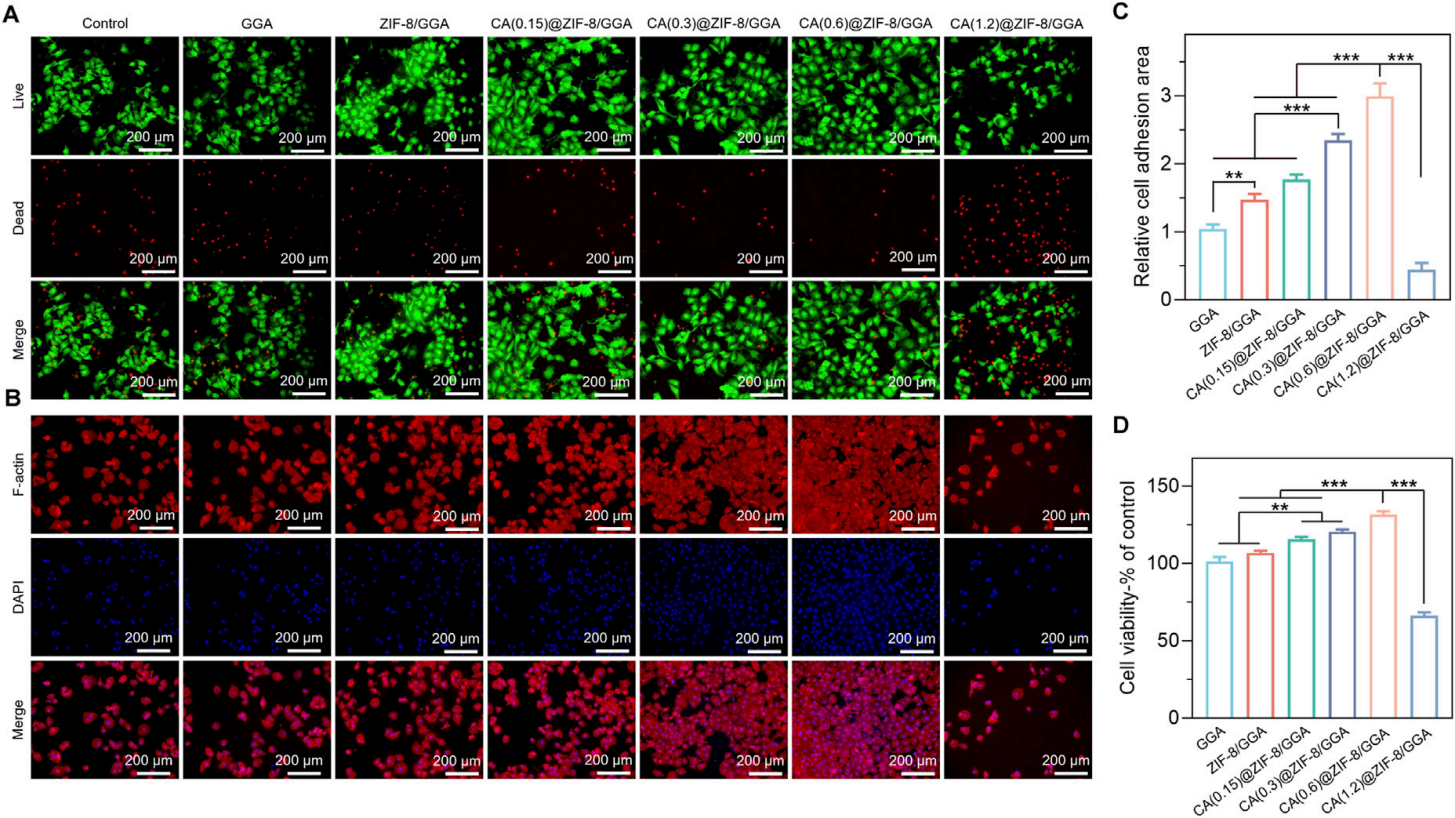
*In vitro* biocompatibility evaluation of GGA, ZIF-8/GGA, and CA(0.15, 0.3, 0.6, 1.2)@ZIF-8/GGA hydrogels with HUVECs. **(A)** Fluorescence images of HUVECs cultured on different hydrogels, including live/dead staining (live cells: green; dead cells: red), and F-actin (red) and nuclei (blue, DAPI) staining. **(B)** Quantification of cell viability of different hydrogels. **(C)** Quantification of relative cell adhesion numbers based on F-actin and DAPI staining Scale bar = 200 μm. **(D)** Quantification of cell viability of HUVECs cultured on different hydrogels, expressed as percentage relative to the control group. Data are presented as mean ± SD (n = 3); *p < 0.05, **p < 0.01, ***p < 0.001.

Live/dead staining of HUVECs (Figure 5A) showed that the GGA hydrogel alone did not enhance cell survival compared to the control group. Incorporation of ZIF-8 modestly improved viability, which was further enhanced when CA was loaded into ZIF-8. A concentration-dependent effect was observed, with the CA(0.6)@ZIF-8/GGA group displaying the highest number of live cells and the fewest dead cells, whereas excessive CA in CA(1.2)@ZIF-8/GGA resulted in significant cytotoxicity. Quantitative analysis of fluorescence intensity (Supplementary Figure S1) confirmed these observations: CA(0.6)@ZIF-8/GGA exhibited the strongest calcein-AM (live) signal and reduced PI (dead) signal, while CA(1.2)@ZIF-8/GGA showed the opposite trend.

F-actin/DAPI staining (Figure 5B) further supported these findings, as cells in the CA(0.6)@ZIF-8/GGA group exhibited optimal adhesion and cytoskeletal organization. Quantification of cell adhesion (Figure 5C) showed the highest relative adhesion area in the CA(0.6)@ZIF-8/GGA group, which was consistent with enhanced spreading. The CCK-8 assay (Figure 5D) also confirmed the superior viability of this group, while CA(1.2)@ZIF-8/GGA again showed a sharp reduction in cell survival. Collectively, these results demonstrate that CA(0.6)@ZIF-8/GGA provides the most favorable cytocompatibility, and thus it was selected for subsequent angiogenesis-related experiments.

### 3.6 Immunofluorescence analysis of angiogenic markers in HUVECs

**FIGURE 6:**
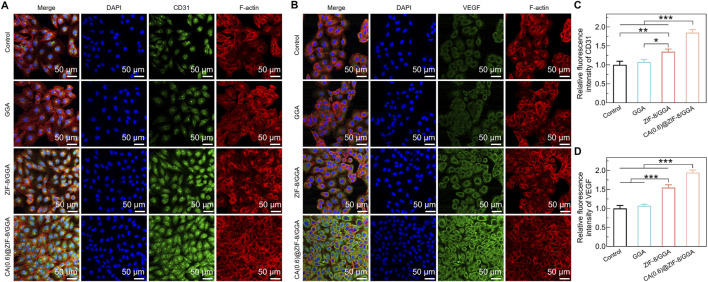
Immunofluorescence analysis of angiogenic markers in HUVECs cultured with hydrogel extracts. **(A)** Representative immunofluorescence images of HUVECs stained for CD31 (green), F-actin (red), and nuclei (DAPI, blue). **(B)** Representative images of VEGF expression (green) with F-actin (red) and DAPI (blue). Scale bar = 50 μm for all images. Primary antibodies (anti-CD31 and anti-VEGF, Abcam) were used at 1:200 dilution, and identical exposure times were applied to all groups to ensure comparability. **(C)** Quantification of relative fluorescence intensity of CD31, normalized to control = 1.0. **(D)** Quantification of relative fluorescence intensity of VEGF, normalized to control = 1.0. Data are presented as mean ± SD (n = 3). Statistical analysis was performed using one-way ANOVA with Tukey’s *post hoc* test; *p < 0.05, **p < 0.01, ***p < 0.001.

To further evaluate the angiogenic potential of the hydrogels, immunofluorescence staining was performed to assess the expression of two key markers: CD31 and VEGF in HUVECs cultured with different hydrogel extracts. As shown in Figure 6A, the expression of CD31 in the control and GGA groups was relatively weak, while ZIF-8/GGA treatment led to moderately enhanced CD31 staining. Notably, CA(0.6)@ZIF-8/GGA treatment resulted in a marked increase in CD31 signal, accompanied by improved cytoskeletal organization and cell spreading. Quantitative analysis (Figure 6C) confirmed a statistically significant upregulation of CD31 in the CA(0.6)@ZIF-8/GGA group compared to the other groups. Figure 6B demonstrates the expression of VEGF, another critical angiogenic factor. A similar trend was observed, with the CA(0.6)@ZIF-8/GGA group showing the highest VEGF fluorescence intensity. Quantification (Figure 6D) revealed a significant increase in VEGF levels, particularly in the CA(0.6)@ZIF-8/GGA group (***p < 0.001), indicating that the composite hydrogel synergistically enhances angiogenic signaling via upregulation of both structural (CD31) and secretory (VEGF) markers.

### 3.7 CA(0.6)@ZIF-8/GGA hydrogel promotes HUVEC migration and tube formation *in vitro*

**FIGURE 7:**
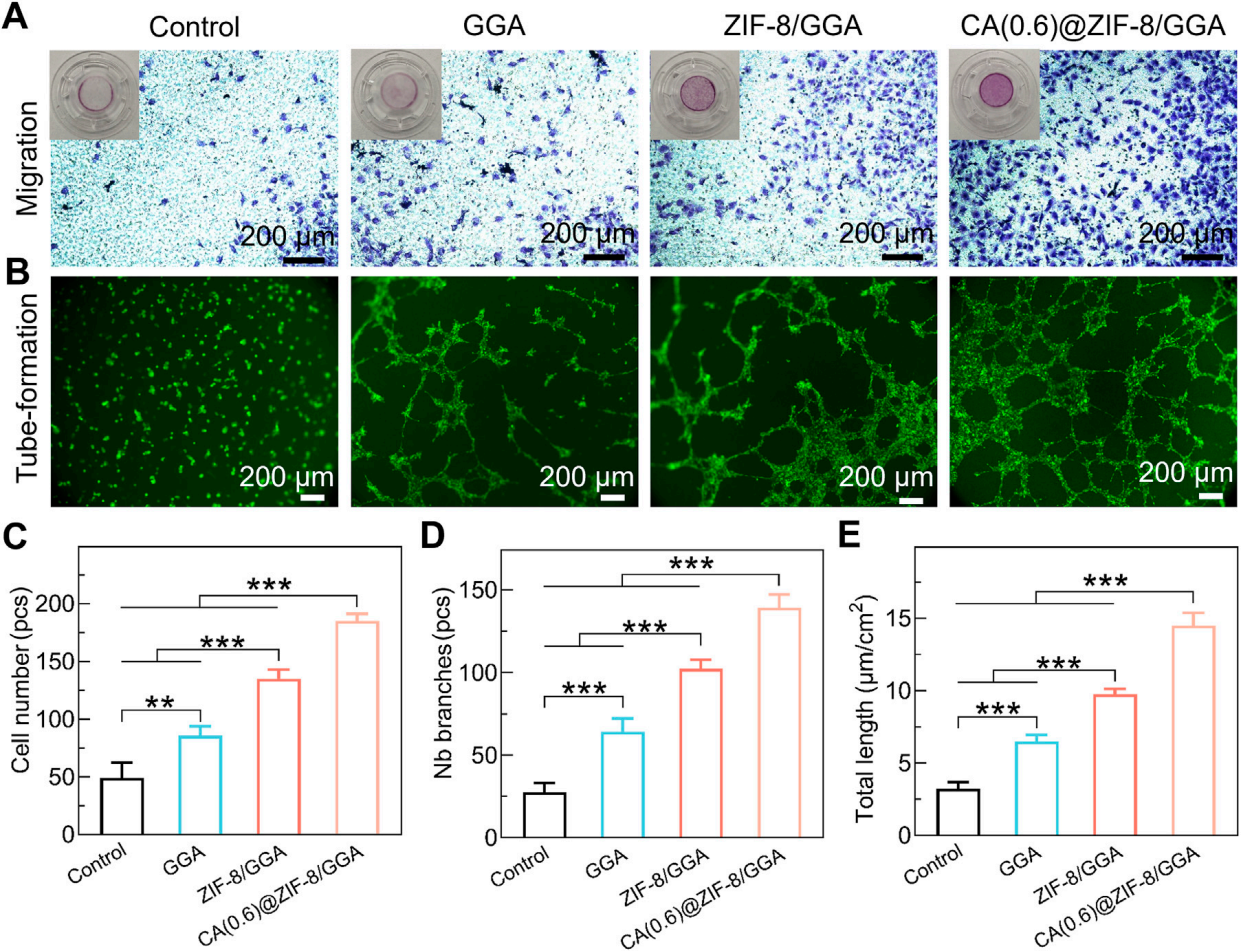
*In vitro* pro-angiogenic activity of hydrogels. **(A)** Transwell migration assay of HUVECs after 24 h culture with hydrogel extracts. Representative images show migrated cells stained with crystal violet. Insets display the corresponding whole Transwell wells for context. Scale bar = 200 μm. **(B)** Tube formation assay of HUVECs cultured on Matrigel in the presence of hydrogel extracts, stained with calcein-AM. Scale bar = 200 μm. **(C)** Quantification of migrated cell number (pcs). **(D)** Quantification of tube formation by number of branches (pcs). **(E)** Quantification of total tube length (µm/cm^2^). Data are presented as mean ± SD (n = 3 independent experiments). Statistical analysis was performed using one-way ANOVA with Tukey’s *post hoc* test; **p < 0.01, ***p < 0.001.

To further assess the proangiogenic capacity of the CA(0.6)@ZIF-8/GGA hydrogel, we performed *in vitro* HUVEC migration and tube formation assays. In the Transwell migration assay (Figure 7A), HUVECs treated with CA(0.6)@ZIF-8/GGA extracts exhibited the highest number of migrated cells compared to the other groups. Quantitative analysis (Figure 7C) showed a ∼3.5-fold increase in migrated cell number relative to the control group (**p < 0.01, ***p < 0.001), suggesting that the composite hydrogel strongly enhances endothelial motility. In the Matrigel tube formation assay (Figure 7B), CA(0.6)@ZIF-8/GGA stimulated the formation of well-defined, interconnected tubular networks with significantly more branches and longer overall tube length compared to the GGA and ZIF-8/GGA groups. Quantification revealed marked increases in both the number of branches (Figure 7D) and total tube length (Figure 7E), with CA(0.6)@ZIF-8/GGA outperforming all other treatments (***p < 0.001). These results corroborate the immunofluorescence findings and confirm that CA(0.6)@ZIF-8/GGA hydrogel effectively promotes angiogenesis-related processes through synergistic regulation of endothelial behavior.

### 3.8 CA(0.6)@ZIF-8/GGA hydrogel significantly accelerates wound closure in a diabetic mouse model

**FIGURE 8:**
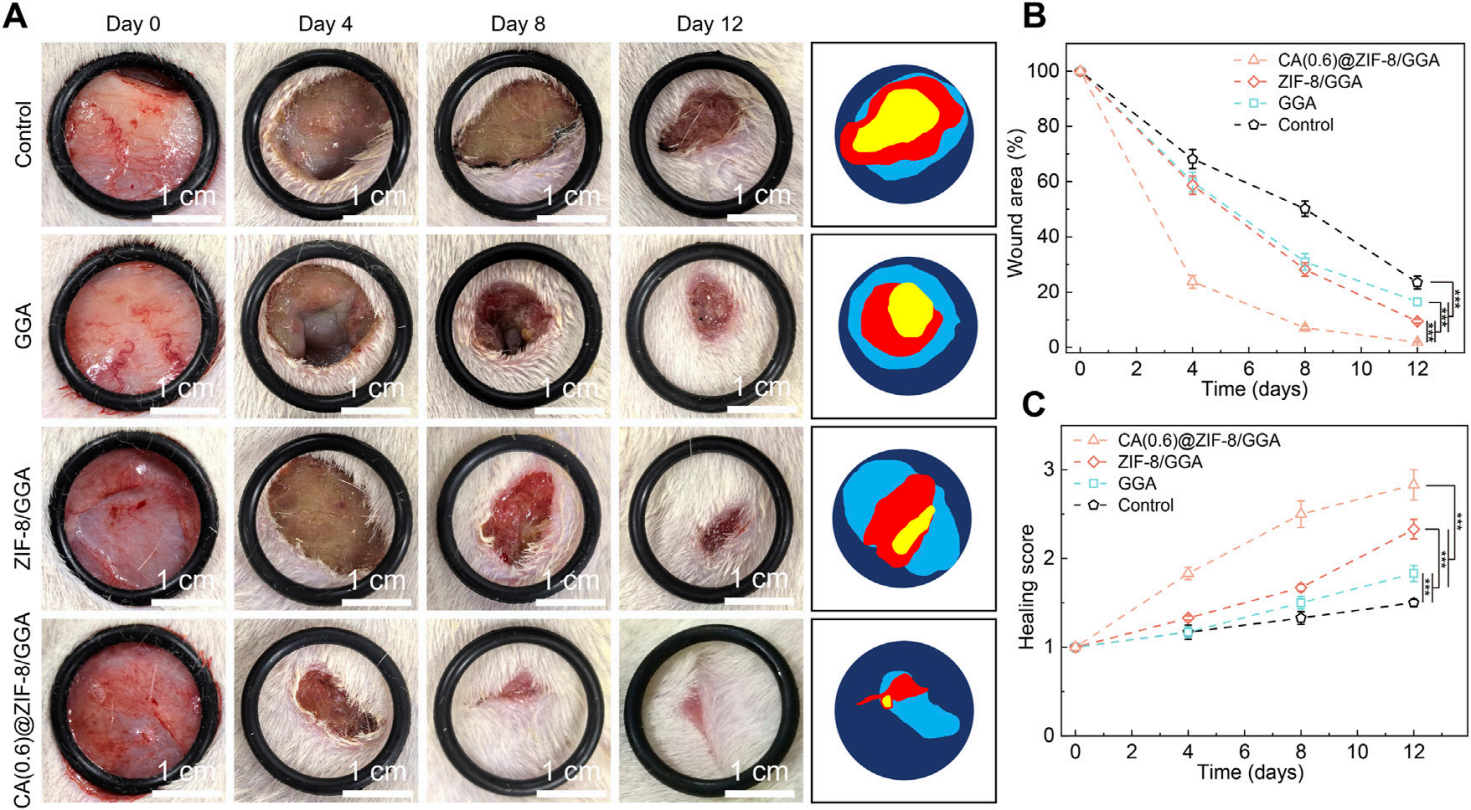
*In vivo* wound healing evaluation in a diabetic rat model. **(A)** Representative wound images of different treatment groups (Control, GGA, ZIF-8/GGA, and CA(0.6)@ZIF-8/GGA) at days 0, 4, 8, and 12, with schematic illustrations of wound areas on the right. Scale bar = 1 cm. All images were taken under consistent lighting conditions. **(B)** Quantification of wound closure, expressed as percentage of the original wound area (%). **(C)** Wound healing score evaluated on a 0–4 scale based on wound edge contraction, granulation tissue formation, and re-epithelialization (detailed criteria provided in Supplementary Table S1). Data are presented as mean ± SD (n = 5 per group). Statistical analysis was performed using one-way ANOVA with Tukey’s *post hoc* test; ***p < 0.001.

To investigate the therapeutic efficacy of the CA(0.6)@ZIF-8/GGA hydrogel *in vivo*, a full-thickness excisional wound model was established in streptozotocin-induced diabetic mice. Wounds were treated with PBS (control), GGA, ZIF-8/GGA, or CA(0.6)@ZIF-8/GGA, and monitored over 12 days. As shown in Figure 8A, wound healing was notably faster in the CA(0.6)@ZIF-8/GGA group, with almost complete re-epithelialization and hair regrowth by day 12. In contrast, the control and GGA groups displayed delayed wound closure and prominent scab formation. Quantitative analysis of wound area (Figure 8B) revealed that CA(0.6)@ZIF-8/GGA significantly reduced wound size from day 4 onwards, achieving over 90% closure by day 12 (***p < 0.001). The healing score, which integrates wound appearance, epithelialization, and contraction, was also highest in the CA(0.6)@ZIF-8/GGA group (Figure 8C). These results suggest that the multifunctional composite hydrogel markedly promotes tissue regeneration and wound closure in diabetic conditions, likely through coordinated regulation of inflammation and angiogenesis.

### 3.9 CA(0.6)@ZIF-8/GGA hydrogel exhibits good systemic biocompatibility *in vivo*

**FIGURE 9:**
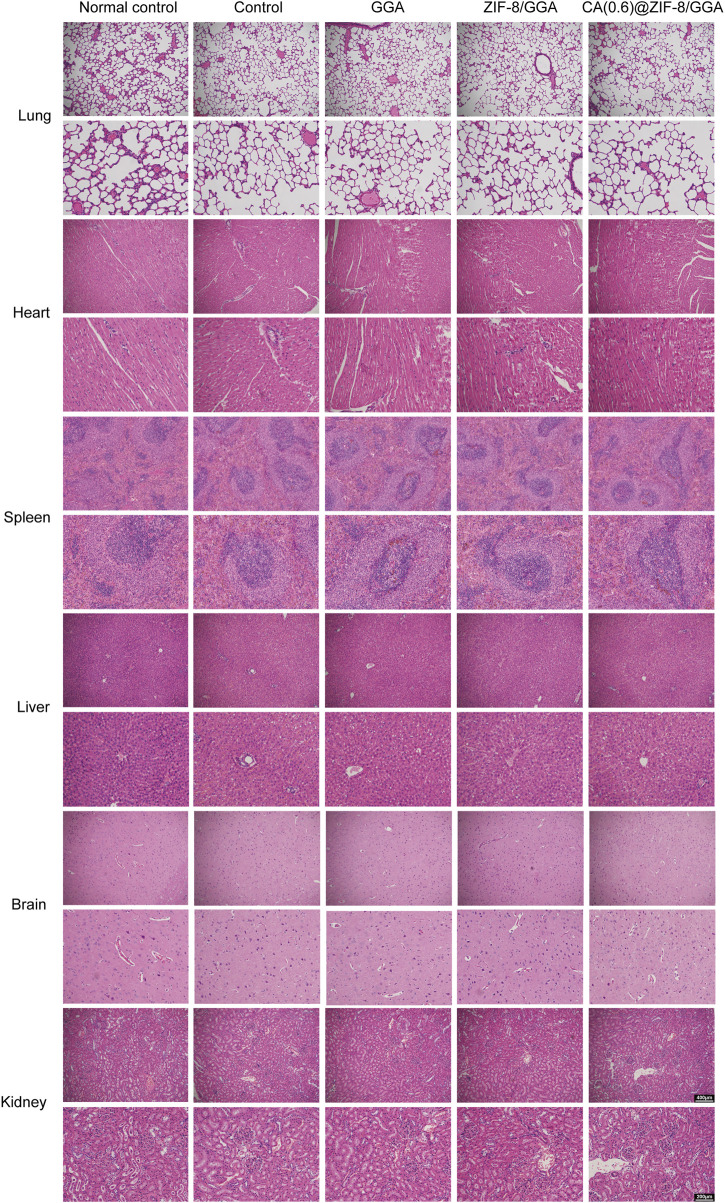
Histological evaluation of major organs to assess systemic biosafety of hydrogel treatments. Representative hematoxylin and eosin (H&E) staining images of lung, liver, heart, brain, spleen, and kidney tissues harvested from mice 12 days after topical application of different hydrogel formulations. No significant histopathological abnormalities or inflammatory infiltrates were observed in any group. Scale bar = 200 μm. Histological evaluation of major organs after hydrogel treatment. Representative H&E staining images of lung, heart, spleen, liver, brain, and kidney tissues collected on day 12 from five groups: Normal control (non-diabetic baseline), diabetic Control (untreated), GGA, ZIF-8/GGA, and CA(0.6)@ZIF-8/GGA. Images were captured at 20× and 40× magnifications, with scale bars = 400 μm (upper panels) and 200 μm (lower panels). Insets highlight key tissue features including alveolar structure, myocardial fibers, splenic follicles, hepatic lobules, neuronal cells, and renal glomeruli/tubules. Histological evaluation was performed in a blinded manner by a trained pathologist, who confirmed the absence of significant histopathological abnormalities across all treatment groups compared to controls.

To assess systemic biosafety, major organs (lung, liver, heart, brain, spleen, and kidney) were collected at day 12 and subjected to H&E staining (Figure 9). All groups, including the CA(0.6)@ZIF-8/GGA-treated rats, exhibited normal histoarchitecture comparable to the normal and diabetic controls. The lung tissues showed intact alveolar structures without hemorrhage or inflammation; liver and kidney sections maintained normal hepatocyte and glomerular morphology; and no myocardial degeneration, neuronal damage, or splenic abnormalities were observed. These findings confirm that topical application of CA(0.6)@ZIF-8/GGA hydrogel does not induce detectable systemic toxicity, supporting its favorable biosafety profile for diabetic wound healing applications.

## 4 Discussion

Diabetic wounds are characterized by persistent inflammation, impaired angiogenesis, and oxidative stress, which collectively hinder tissue regeneration and lead to chronic non-healing ulcers. In this study, we constructed an injectable composite hydrogel by integrating cinnamaldehyde-loaded ZIF-8 nanoparticles (CA@ZIF-8) into a GGA matrix. This multifunctional hydrogel exhibited tunable mechanical properties, sustained drug release, and pro-angiogenic activity, ultimately accelerating wound healing in a diabetic rat model. Compared with traditional dressings or single-component hydrogels, our system achieves synergistic therapeutic effects through the combination of polyphenols, MOFs, and bioactive aldehydes.

GA is a naturally occurring polyphenol with potent antioxidant and mild anti-inflammatory properties. Previous studies have shown that GA-modified biomaterials can effectively scavenge ROS, reduce matrix metalloproteinase activity, and promote cellular migration and proliferation (Yang et al., 2023a; Chen et al., 2024). For instance, Wang et al. reported that gallic acid-modified chitosan hydrogel significantly accelerated full-thickness wound healing by modulating oxidative stress and enhancing fibroblast proliferation (Sheng et al., 2023). In another study, a GA-functionalized hyaluronic acid hydrogel showed enhanced endothelial cell tube formation and collagen deposition *in vivo* (Yang et al., 2023b).

However, most reported GA-based hydrogels rely on relatively weak hydrogen bonding or Schiff base crosslinking, which limits their long-term mechanical stability. In our system, GA was covalently grafted onto gelatin, enabling phenol-metal coordination and transglutaminase (TGase)–mediated enzymatic crosslinking. This strategy not only improves hydrogel stiffness and durability, but also facilitates gradual Zn^2+^ release from the embedded ZIF-8 framework. Moreover, the phenolic moieties from GA may chelate with Zn^2+^ to form additional dynamic bonds, further contributing to the integrity of the hydrogel network.

Gelatin is one of the most widely used natural polymers in wound dressings due to its excellent biocompatibility, biodegradability, and ability to mimic extracellular matrix (ECM) components. Multiple studies have confirmed the feasibility of gelatin-based injectable hydrogels in diabetic wound healing. For instance, Shen et al. developed a gelatin–boronic acid hydrogel that responded to glucose concentration in diabetic wounds and facilitated fibroblast migration and vascularization (Liu et al., 2024). Similarly, Zhang et al. reported a gelatin–EGCG injectable hydrogel that enhanced antioxidant activity and promoted re-epithelialization (Chang et al., 2016).

Compared with these systems, our GGA-based hydrogel exhibits additional phenol-rich functionality and better mechanical performance. More importantly, the use of TGase crosslinking provides a mild, cytocompatible route to gel formation, allowing *in situ* injection into irregular wound sites without requiring external stimuli or high temperature. The resulting hydrogel forms a conformal barrier that supports cellular infiltration, maintains hydration, and enables sustained release of incorporated therapeutics.

CA, a bioactive compound derived from cinnamon bark, has garnered increasing attention for its antibacterial, antioxidant, and pro-angiogenic properties. Several studies have reported that CA can inhibit bacterial biofilm formation, scavenge ROS, and regulate angiogenic signaling pathways such as VEGF and HIF-1α (Tu et al., 2022; Luo et al., 2021). For example, Zhao et al. developed a CA-loaded electrospun nanofiber membrane that accelerated diabetic wound healing by promoting vascularization and reducing oxidative stress (Liu et al., 2017). Yang et al. demonstrated that CA enhances endothelial cell migration and tube formation by activating PI3K/Akt signaling (Yan et al., 2024).

However, the major limitation of CA lies in its volatility and poor water solubility, which restricts its bioavailability in aqueous environments. Our use of ZIF-8 as a pH-responsive carrier not only protects CA from premature degradation but also allows controlled, sustained release under the slightly acidic conditions of the diabetic wound environment. This approach significantly improves CA’s therapeutic efficacy. Additionally, the synergistic presence of Zn^2+^ from ZIF-8 further amplifies the pro-angiogenic response.

Our *in vitro* experiments demonstrated that CA(0.6)@ZIF-8/GGA significantly enhanced HUVEC viability, migration, and tube formation, along with elevated CD31 and VEGF expression. These results are consistent with the known roles of CA and Zn^2+^ in promoting endothelial cell activation. Importantly, the optimal concentration of CA (0.6 mg/mL) achieved a balance between pro-angiogenic stimulation and cytocompatibility. In contrast, a higher loading CA(1.2)@ZIF-8/GGA led to reduced cell viability, highlighting a concentration-dependent effect. Previous studies have reported that elevated CA levels can disrupt cell membrane integrity, increase intracellular reactive oxygen species (ROS), and induce mitochondrial dysfunction, ultimately triggering apoptosis. When combined with ZIF-8, excessive CA may also accelerate Zn^2+^ release, further aggravating cytotoxic stress. These findings underscore the importance of dosage optimization to maximize therapeutic efficacy while minimizing potential toxicity. In the diabetic rat model, CA@ZIF-8/GGA-treated wounds exhibited faster closure, increased granulation tissue formation, and enhanced collagen deposition. Histological staining further confirmed significantly improved neovascularization, while no pathological abnormalities were detected in major organs, supporting the hydrogel’s biosafety. Compared with previous works utilizing growth factors, which are expensive and prone to degradation, our hydrogel system relies on small-molecule bioactivity and MOF-assisted delivery, providing a more cost-effective and stable solution for clinical translation.

Although this study highlights the potential of the CA@ZIF-8/GGA hydrogel for diabetic wound healing, some limitations remain. First, while angiogenesis was thoroughly assessed, inflammation-related pathways were not directly investigated in this study. Our previous work has demonstrated that GA- and CA-based hydrogels can modulate macrophage polarization and suppress NF-κB–mediated inflammation (Li G. et al., 2023; Deng et al., 2023), but these effects were not experimentally validated here. Future studies should include analyses of inflammatory cytokines (e.g., IL-6, TNF-α), macrophage markers (e.g., CD86, CD206), and relevant signaling pathways (e.g., TLR4/NF-κB). Second, long-term evaluations of wound remodeling, including hair follicle regeneration, skin appendage formation, and scar formation, are necessary to fully assess the functional recovery of the regenerated skin. Furthermore, hydrogel degradation products and their interactions with local immune cells warrant systematic investigation.

## 5 Conclusion

In conclusion, we developed a rationally designed injectable hydrogel platform that combines GGA with CA@ZIF-8 to address the dual challenges of vascular deficiency and chronic inflammation in diabetic wounds. This multifunctional system exhibited favorable mechanical integrity, co-delivery of CA and Zn^2+^, and potent pro-angiogenic activity *in vitro*. In streptozotocin-induced diabetic rats, the optimized CA(0.6)@ZIF-8/GGA hydrogel accelerated wound closure, enhanced neovascularization, and improved granulation tissue formation compared to other groups, highlighting its therapeutic efficacy. Nevertheless, this study has limitations, including the lack of long-term evaluation of wound recurrence and incomplete elucidation of inflammation-related mechanisms. Future work will focus on extended *in vivo* assessments, detailed mechanistic studies, and optimization of large-scale, reproducible synthesis to facilitate clinical translation. Overall, the CA@ZIF-8/GGA hydrogel offers a promising and versatile therapeutic strategy for chronic wound management with high translational potential.

## Data Availability

The raw data supporting the conclusions of this article will be made available by the authors, without undue reservation.

## References

[B1] Chang C. Y. Wang M. C. Miyagawa T. Chen Z. Y. Lin F. H. Chen K. H. (2016). Preparation of arginine-glycine-aspartic acid-modified biopolymeric nanoparticles containing epigalloccatechin-3-gallate for targeting vascular endothelial cells to inhibit corneal neovascularization. Int. J. Nanomedicine 12, 279–294. 10.2147/IJN.S114754 28115846 PMC5221810

[B2] Chen Q. Qian Q. Xu H. Zhou H. Chen L. Shao N. (2024). Mitochondrial-Targeted metal-phenolic nanoparticles to attenuate intervertebral disc degeneration: alleviating oxidative stress and mitochondrial dysfunction. ACS Nano 18 (12), 8885–8905. 10.1021/acsnano.3c12163 38465890

[B3] Deng J. Liu S. Li G. Zheng Y. Zhang W. Lin J. (2023). pH-sensitive charge-conversion cinnamaldehyde polymeric prodrug micelles for effective targeted chemotherapy of osteosarcoma *in vitro* . Front. Chem. 11, 1190596. 10.3389/fchem.2023.1190596 37206197 PMC10188981

[B4] Ding B. Chen H. Tan J. Meng Q. Zheng P. Ma P. (2023). ZIF-8 nanoparticles evoke pyroptosis for high-efficiency cancer immunotherapy. Angew. Chem. Int. Ed. Engl. 62 (10), e202215307. 10.1002/anie.202215307 36629270

[B5] Ge Y. Wang K. Liu J. Tian Y. Li H. Wang H. (2022). A ZIF-8-based multifunctional intelligent drug release system for chronic osteomyelitis. Colloids Surf. B Biointerfaces 212, 112354. 10.1016/j.colsurfb.2022.112354 35085938

[B6] Guan J. Wang X. Tian Z. Jia F. Wang J. Xie L. (2025). Controlled-release of cinnamaldehyde from MXene/ZIF8/gelatin composite coatings: an integrated strategy to combat implant-associated infection. Colloids Surf. B Biointerfaces 251, 114615. 10.1016/j.colsurfb.2025.114615 40086209

[B7] Li Y. Zhu J. Zhang X. Li Y. Zhang S. Yang L. (2023a). Drug-delivery nanoplatform with synergistic regulation of angiogenesis-osteogenesis coupling for promoting vascularized bone regeneration. ACS Appl. Mater Interfaces 15 (14), 17543–17561. 10.1021/acsami.2c23107 37010447

[B8] Li G. Liu S. Chen Y. Zhao J. Xu H. Weng J. (2023b). An injectable liposome-anchored teriparatide incorporated gallic acid-grafted gelatin hydrogel for osteoarthritis treatment. Nat. Commun. 14 (1), 3159. 10.1038/s41467-023-38597-0 37258510 PMC10232438

[B9] Liang Y. He J. Guo B. (2021). Functional hydrogels as wound dressing to enhance wound healing. ACS Nano 15 (8), 12687–12722. 10.1021/acsnano.1c04206 34374515

[B10] Liu Y. Liang X. Zhang R. Lan W. Qin W. (2017). Fabrication of electrospun polylactic Acid/Cinnamaldehyde/β-Cyclodextrin fibers as an antimicrobial wound dressing. Polym. (Basel) 9 (10), 464. 10.3390/polym9100464 30965767 PMC6418790

[B11] Liu L. Wang W. Huang L. Xian Y. Ma W. Fan J. (2024). Injectable pathological microenvironment-responsive anti-inflammatory hydrogels for ameliorating intervertebral disc degeneration. Biomaterials 306, 122509. 10.1016/j.biomaterials.2024.122509 38377847

[B12] Liu Y. Yang G. Liu M. Zhang Y. Xu H. Mazhar M. (2025). Cinnamaldehyde and its combination with deferoxamine ameliorate inflammation, ferroptosis and hematoma expansion after intracerebral hemorrhage in mice. J. Neuroinflammation 22 (1), 45. 10.1186/s12974-025-03373-y 39985048 PMC11846400

[B13] Luo X. Zhao B. Chen B. Chen H. Han T. Bsoul N. B. N. (2021). Trans-cinnamaldehyde increases random pattern flap survival through activation of the nitric oxide pathway. Drug Des. Devel Ther. 15, 679–688. 10.2147/DDDT.S297458 33628013 PMC7899309

[B14] Mallawarachchi S. Mahadevan A. Gejji V. Fernando S. (2019). Mechanics of controlled release of insulin entrapped in polyacrylic acid gels via variable electrical stimuli. Drug Deliv. Transl. Res. 9 (4), 783–794. 10.1007/s13346-019-00620-7 30767123

[B15] Pan W. Qi X. Xiang Y. You S. Cai E. Gao T. (2022). Facile formation of injectable quaternized chitosan/tannic acid hydrogels with antibacterial and ROS scavenging capabilities for diabetic wound healing. Int. J. Biol. Macromol. 195, 190–197. 10.1016/j.ijbiomac.2021.12.007 34896467

[B16] Qian J. Zhang W. Chen Y. Zeng P. Wang J. Zhou C. (2022). Osteogenic and angiogenic bioactive collagen entrapped calcium/zinc phosphates coating on biodegradable Zn for orthopedic implant applications. Biomater. Adv. 136, 212792. 10.1016/j.bioadv.2022.212792 35929323

[B17] Qian J. Qin H. Zeng P. Hou J. Mo X. Shen G. (2023). Metal-organic Zn-zoledronic acid and 1-hydroxyethylidene-1,1-diphosphonic acid nanostick-mediated zinc phosphate hybrid coating on biodegradable Zn for osteoporotic fracture healing implants. Acta Biomater. 166, 685–704. 10.1016/j.actbio.2023.05.020 37196904

[B18] Shao Z. Yin T. Jiang J. He Y. Xiang T. Zhou S. (2022). Wound microenvironment self-adaptive hydrogel with efficient angiogenesis for promoting diabetic wound healing. Bioact. Mater 20, 561–573. 10.1016/j.bioactmat.2022.06.018 35846841 PMC9254353

[B19] Sheng W. Qin H. Wang T. Zhao J. Fang C. Zhang P. (2023). Advanced phosphocreatine-grafted chitosan hydrogel promote wound healing by macrophage modulation. Front. Bioeng. Biotechnol. 11, 1199939. 10.3389/fbioe.2023.1199939 37251563 PMC10213409

[B20] Tang L. Zhang Z. Lei S. Zhou J. Liu Y. Yu X. (2023). A temperature and pH dual-responsive injectable self-healing hydrogel prepared by chitosan oligosaccharide and aldehyde hyaluronic acid for promoting diabetic foot ulcer healing. Int. J. Biol. Macromol. 253 (Pt 6), 127213. 10.1016/j.ijbiomac.2023.127213 37793511

[B21] Tu Y. Xiao X. Dong Y. Li J. Liu Y. Zong Q. (2022). Cinnamaldehyde-based poly(thioacetal): a ROS-awakened self-amplifying degradable polymer for enhanced cancer immunotherapy. Biomaterials 289, 121795. 10.1016/j.biomaterials.2022.121795 36108580

[B22] Uccioli L. Izzo V. Meloni M. Vainieri E. Ruotolo V. Giurato L. (2015). Non-healing foot ulcers in diabetic patients: general and local interfering conditions and management options with advanced wound dressings. J. Wound Care 24 (4 Suppl. l), 35–42. 10.12968/jowc.2015.24.Sup4b.35 25853647

[B23] Yan S. Bao S. Chen T. Chen J. Zhang J. Hu X. (2024). Cinnamaldehyde alleviates aspirin-induced gastric mucosal injury by regulating pi3k/akt pathway-mediated apoptosis, autophagy and ferroptosis. Phytomedicine 132, 155791. 10.1016/j.phymed.2024.155791 38901284

[B24] Yang J. Huang Z. Tan J. Pan J. Chen S. Wan W. (2023a). Copper ion/gallic acid MOFs-laden adhesive pomelo peel sponge effectively treats biofilm-infected skin wounds and improves healing quality. Bioact. Mater 32, 260–276. 10.1016/j.bioactmat.2023.10.005 37869725 PMC10589730

[B25] Yang J. Hsu C. C. Cao T. T. Ye H. Chen J. Li Y. Q. (2023b). A hyaluronic acid granular hydrogel nerve guidance conduit promotes regeneration and functional recovery of injured sciatic nerves in rats. Neural Regen. Res. 18 (3), 657–663. 10.4103/1673-5374.350212 36018191 PMC9727441

[B26] Yin X. Wei Y. Qin H. Zhao J. Chen Y. Yao S. (2024). Oxygen tension regulating hydrogels for vascularization and osteogenesis via sequential activation of HIF-1α and ERK1/2 signaling pathways in bone regeneration. Biomater. Adv. 161, 213893. 10.1016/j.bioadv.2024.213893 38796955

[B27] Zeng Q. Li N. Wang Q. Feng J. Sun D. Zhang Q. (2019). The prevalence of osteoporosis in China, a nationwide, multicenter DXA survey. J. Bone Min. Res. 34 (10), 1789–1797. 10.1002/jbmr.3757 31067339

[B28] Zhang J. Xue Y. Zhang L. Chen J. Ma D. Zhang Y. (2025). A targeted core-shell ZIF-8/Au@Fe_3_O_4_ platform with multiple antibacterial pathways for infected skin wound regeneration. ACS Appl. Mater Interfaces 17 (14), 20901–20918. 10.1021/acsami.5c00697 40132060

[B29] Zhao J. Wang T. Zhu Y. Qin H. Qian J. Wang Q. (2024). Enhanced osteogenic and ROS-scavenging MXene nanosheets incorporated gelatin-based nanocomposite hydrogels for critical-sized calvarial defect repair. Int. J. Biol. Macromol. 269 (Pt 1), 131914. 10.1016/j.ijbiomac.2024.131914 38703527

