# Supplementary material for: Injectable cinnamaldehyde–loaded ZIF-8/Gallic Acid–Grafted gelatin hydrogel for enhanced angiogenesis and skin regeneration in diabetic wound healing
Source: Front Bioeng Biotechnol. 2025 Sep 11;13:1660821. doi: 10.3389/fbioe.2025.1660821 (PMC12460342; doi:10.3389/fbioe.2025.1660821)
Supplement: Supplementary file 1 [file DataSheet1.pdf]

Supplementary material

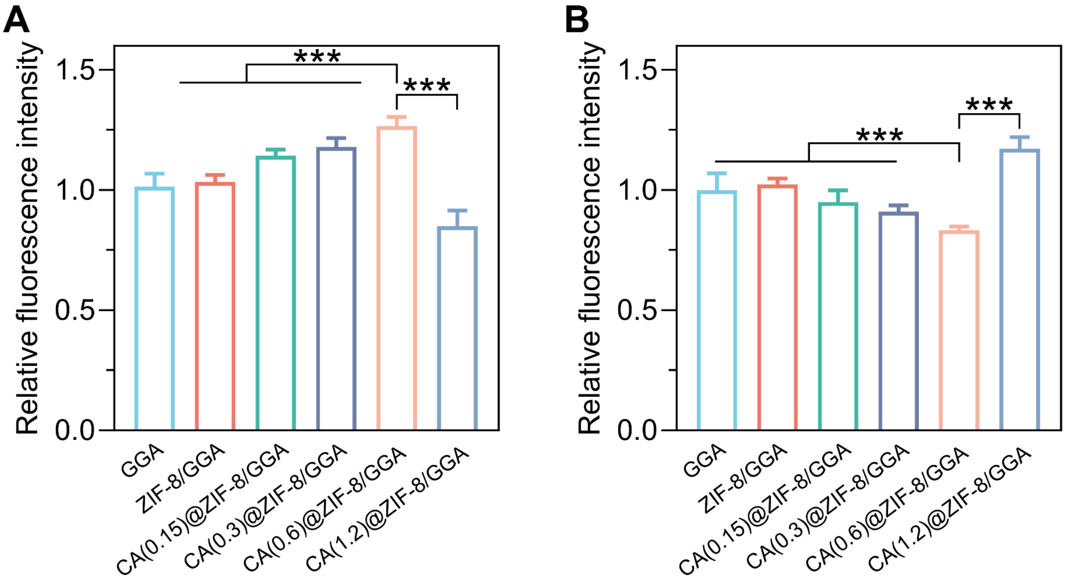

Supplementary Figure S1. Quantitative analysis of HUVEC viability by live/dead staining. (A) Relative fluorescence intensity of live cells (calcein-AM, green) and (B) dead cells (PI, red) after 24 h culture with different hydrogels. Data were normalized to the control group (set as 1.0). The CA(0.6)@ZIF-8/GGA group showed the highest live signal with minimal dead signal, whereas CA(1.2)@ZIF-8/GGA displayed the opposite trend. Data are presented as mean ± SD (n = 3). One-way ANOVA with Tukey's post hoc test; ***$p < 0.001$.

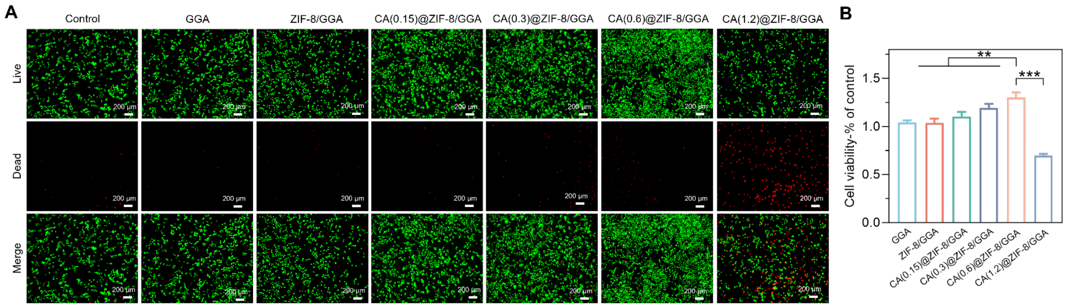

Supplementary Figure S2. Cytocompatibility evaluation of hydrogels with L929 fibroblasts. (A) Live/dead staining images of L929 cells cultured with different hydrogels for 24 h. Live cells were stained green (calcein-AM) and dead cells red (PI). Scale bar = 200 μm. (B) Cell viability quantified by CCK-8 assay after 24 h of culture, expressed as a percentage of the control group. Data are presented as mean ± SD (n = 3). One-way ANOVA with Tukey's test; $^{**}p < 0.01$, $^{***}p < 0.001$.

Table S1. Grading system for wound healing in full-thickness skin wounds.

Grade	Description
0	No closure; open wound with moist surface, obvious exudate; necrotic tissue may be present; no re-epithelialization.
1	≤25% closure; persistent exudate/necrotic tissue; minimal granulation; re-epithelialization absent or very limited.
2	26–50% closure; exudate reduced; partial re-epithelialization at margins; early granulation tissue.
3	51–75% closure; clear re-epithelialization and healthy granulation; minimal exudate; wound largely contracted.
4	≥90% closure; continuous epithelium, dry surface, minimal/no exudate; beginning hair regrowth.
